# Publisher Correction: Nuclear compartmentalization of TERT mRNA and TUG1 lncRNA is driven by intron retention

**DOI:** 10.1038/s41467-021-26492-5

**Published:** 2021-10-21

**Authors:** Gabrijela Dumbović, Ulrich Braunschweig, Heera K. Langner, Michael Smallegan, Josep Biayna, Evan P. Hass, Katarzyna Jastrzebska, Benjamin Blencowe, Thomas R. Cech, Marvin H. Caruthers, John L. Rinn

**Affiliations:** 1 Department of Biochemistry, University of Colorado Boulder, Boulder, CO USA; 2 BioFrontiers Institute, University of Colorado Boulder, Boulder, CO USA; 3 Donnelly Centre, University of Toronto, Ontario, Canada; 4 Department of Molecular, Cellular and Developmental Biology, University of Colorado Boulder, Boulder, CO USA; 5 Institute for Research in Biomedicine, Parc Científic de Barcelona, Barcelona, Spain; 6 Howard Hughes Medical Institute, University of Colorado Boulder, Boulder, CO USA; 7 Present Address: Max Planck Institute of Immunobiology and Epigenetics, Freiburg, Germany; 8 Present Address: Department of Bioorganic Chemistry, Centre of Molecular and Macromolecular Studies, Polish Academy of Sciences, Lodz, Poland

**Keywords:** Cancer, Cell biology, Computational biology and bioinformatics

Correction to: *Nature Communications* 10.1038/s41467-021-23221-w, published online 3 June 2021.

The original version of this Article contained errors in the text (Introduction, Results and Discussion), which incorrectly cited reference 7 in several places. This has been corrected in both the PDF and HTML versions of the Article.

