# Supplementary material for: Nuclear compartmentalization of TERT mRNA and TUG1 lncRNA is driven by intron retention
Source: Nat Commun. 2021 Jun 3;12:3308. doi: 10.1038/s41467-021-23221-w (PMC8175569; doi:10.1038/s41467-021-23221-w)
Supplement: Supplementary file 1 — Supplementary information [file 41467_2021_23221_MOESM1_ESM.pdf]

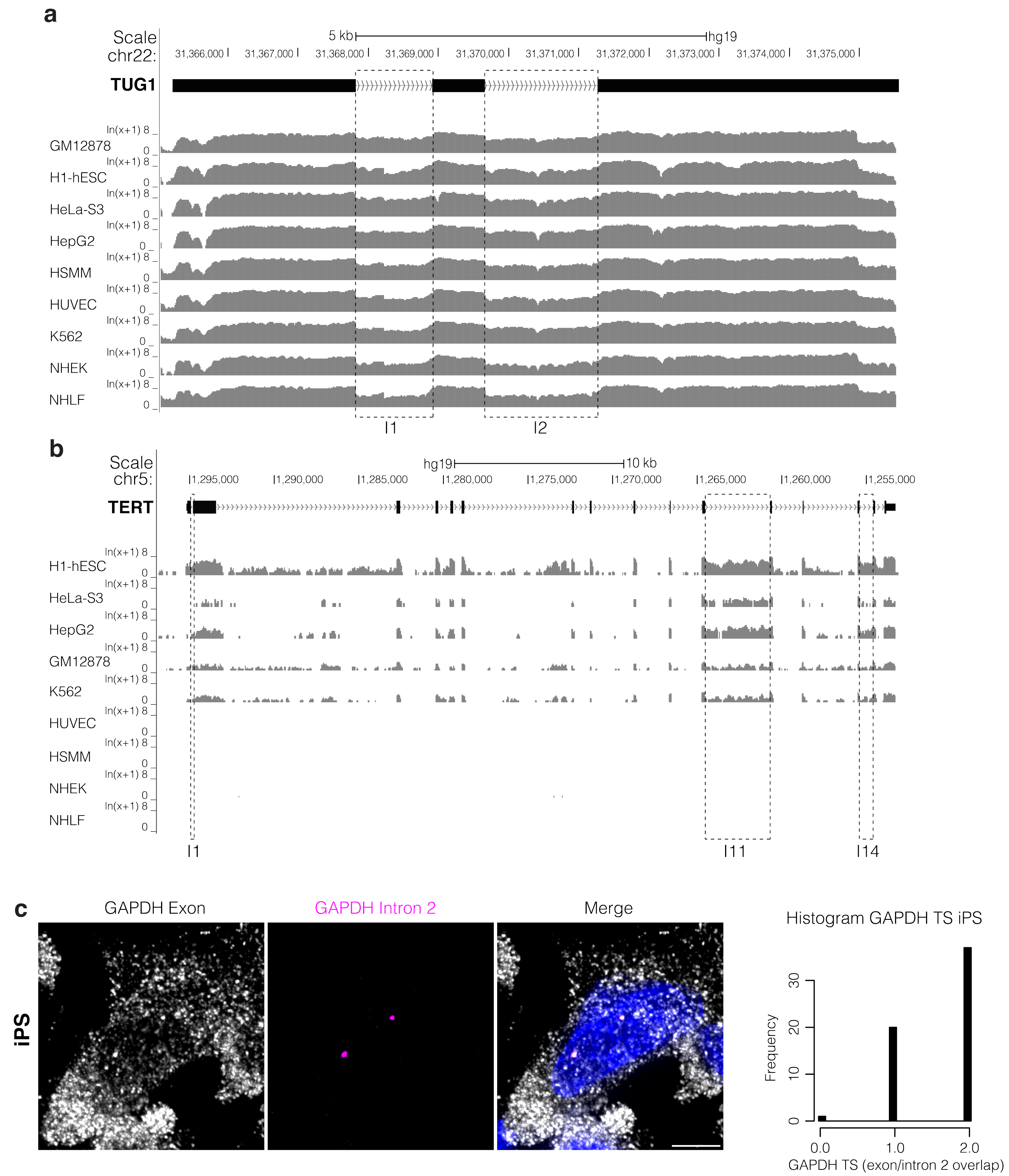

Supplementary figure 1: a, UCSC Genome Browser showing poly(A)+ RNA-seq tracks across the TUG1 locus from indicated cell lines available from ENCODE. **b**, UCSC Genome Browser showing poly(A)+ RNA-seq tracks across the TERT locus from indicated cell lines available from ENCODE. **c**, Maximum intensity projection of GAPDH exon and intron 2 smRNA FISH on iPS cells. Nucleus in blue, exon in gray, intron in magenta. Scale bar, 5 µm. On the right, histogram showing the quantity of exon/intron2 signal overlap in iPS cells. *N* = 58 cells.

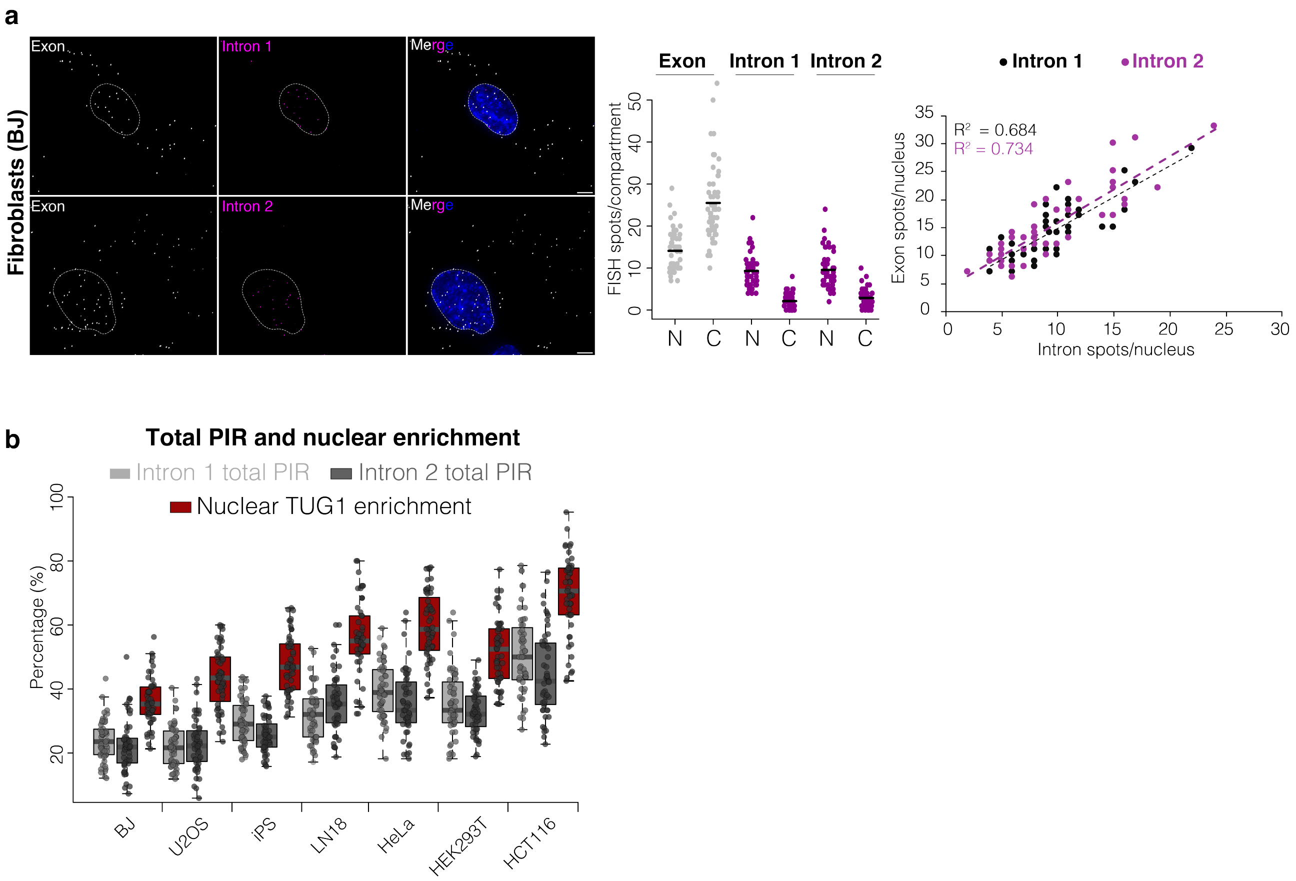

Supplementary figure 2: a, Maximum intensity projections of representative images of TUG1 exon/intron smRNA FISH on human foreskin fibroblasts (BJ). Exon in gray, intron 1 and intron 2 in magenta. Nucleus in blue and outlined with a dashed line. Scale bar, 5 µm. Middle: quantification (*n* = 50 cells) of spliced and unspliced transcripts for each intron in the nucleus (N) and cytoplasm (C), solid line represents the mean. On the right: correlation between nuclear intron count and quantity of nuclear TUG1; intron 1 in black, intron 2 in magenta. **b**, Total percentage of intron retention (total PIR) of each intron and percentage of nuclear enrichment of TUG1 (nuclear TUG1 over total cell TUG1) across indicated cell lines. Each dot represents one cell, *n* = 50 cells, at least 2 independent RNA FISH stainings. Midline line, median; lower and upper box limits, 25th and 75th percentiles; whiskers, 1.5 times interquartile range (IQR) from 25th and 75th percentiles.

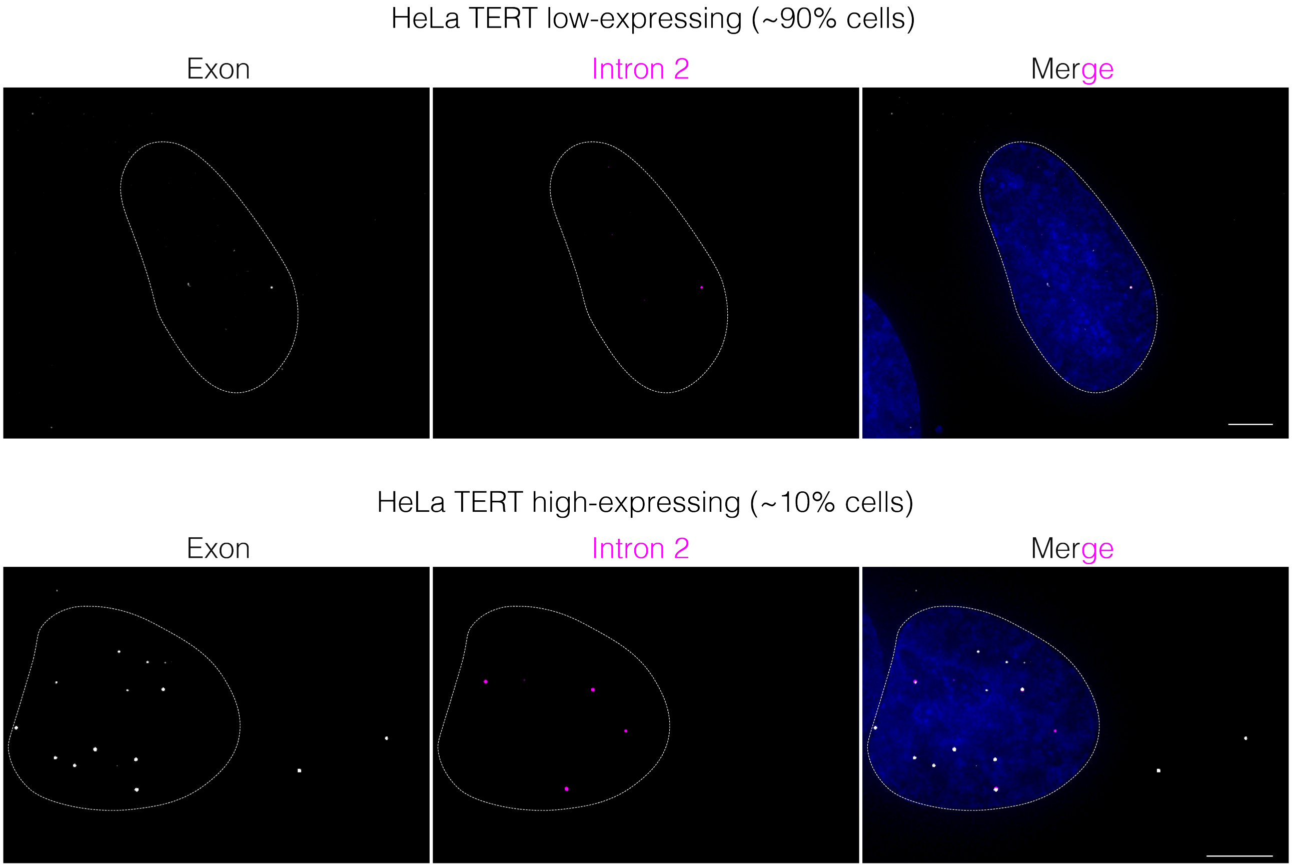

Supplementary figure 3: Maximum intensity projections of representative images of TERT exon and intron 2 smRNA FISH on HeLa cells. Exon in gray, intron 2 in magenta. Nucleus in blue and outlined with a dashed line. Scale bar, 5 µm. On top shown TERT low-expressing cell, on bottom TERT high-expressing cell. Experiment was performed three independent times.

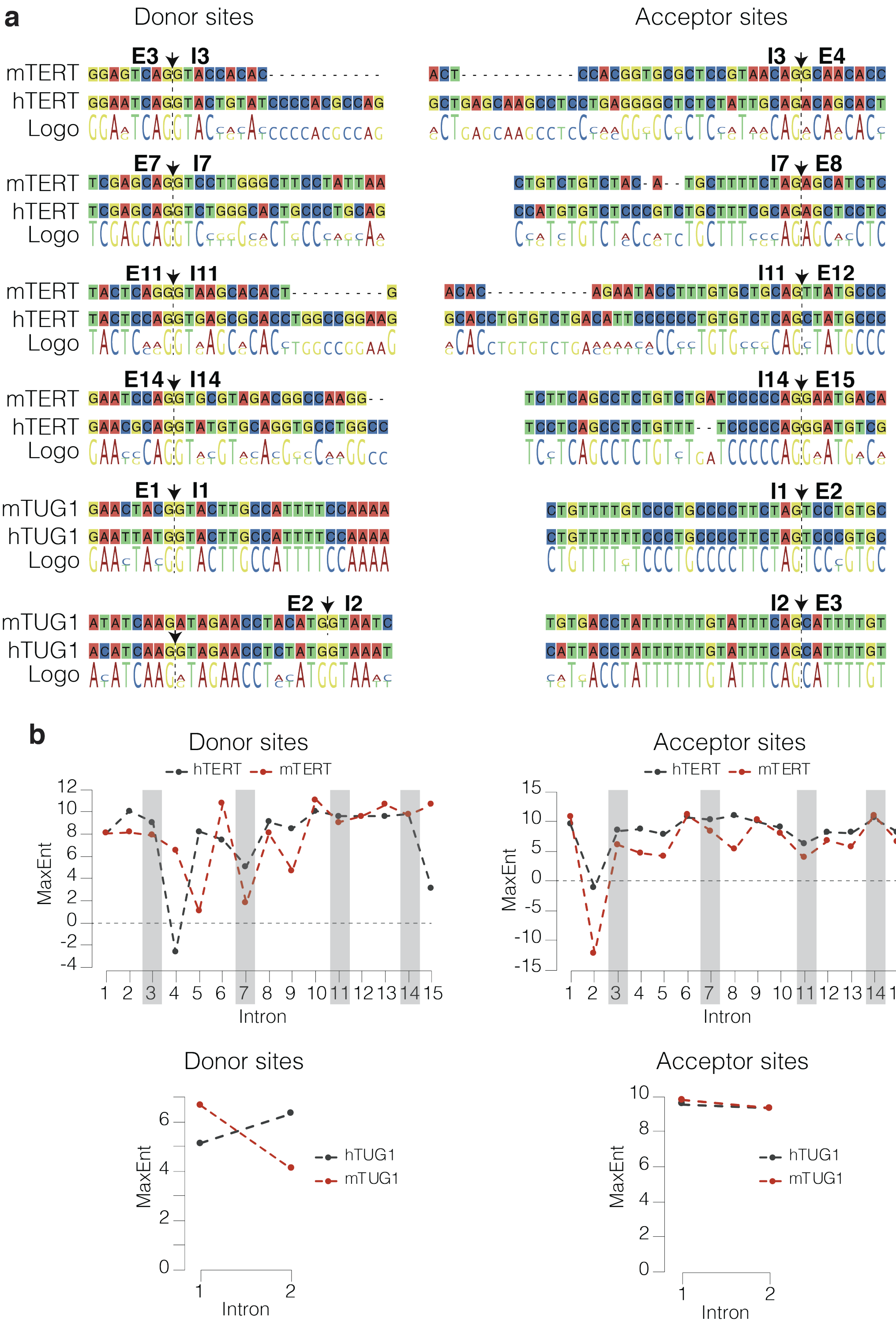

Supplementary figure 4: a, Alignments of donor and acceptor splice sites and surrounding regions between human and mouse TUG1 and TERT. Arrows and dashed lines indicate the splice site. Mouse Tug1 E2/I2 junction is downstream of the E2/I2 junction in human TUG1. **b**, Comparison of donor and acceptor splice site strength measured using maximum entropy (MaxEnt) of human and mouse TUG1 and TERT retained and constitutively spliced introns.

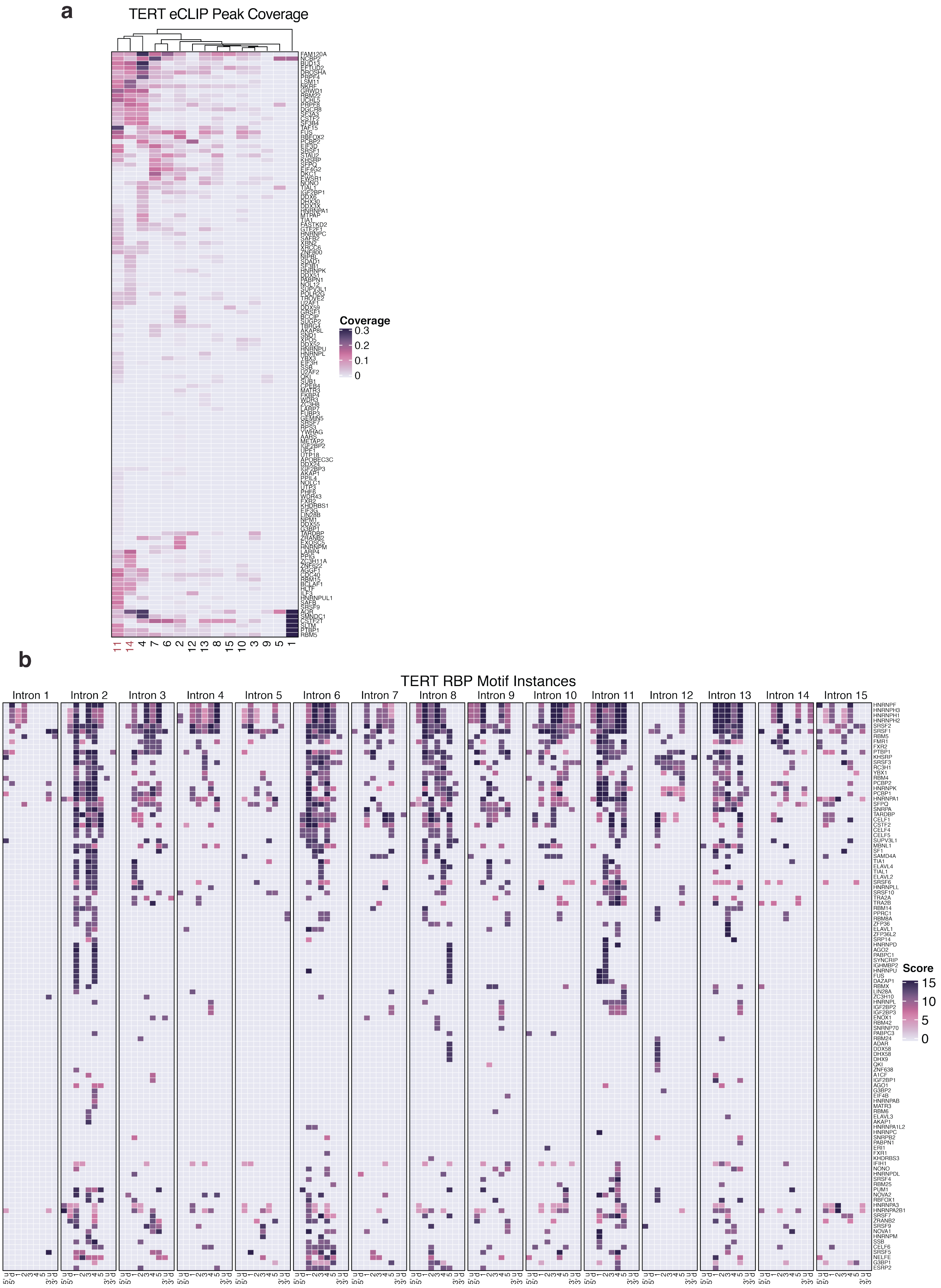

Supplementary figure 5: eCLIP and RBPs motifs across TERT introns. a, Heatmap colored by total intron covered by eCLIP peaks for each of the 127 RBPs with TERT intron binding. **b**, Motif instance matches in TERT introns. In each window, the color represents the maximum scoring motif match in the window as calculated by FIMO (motifs only shown with $p < 1e-4$).

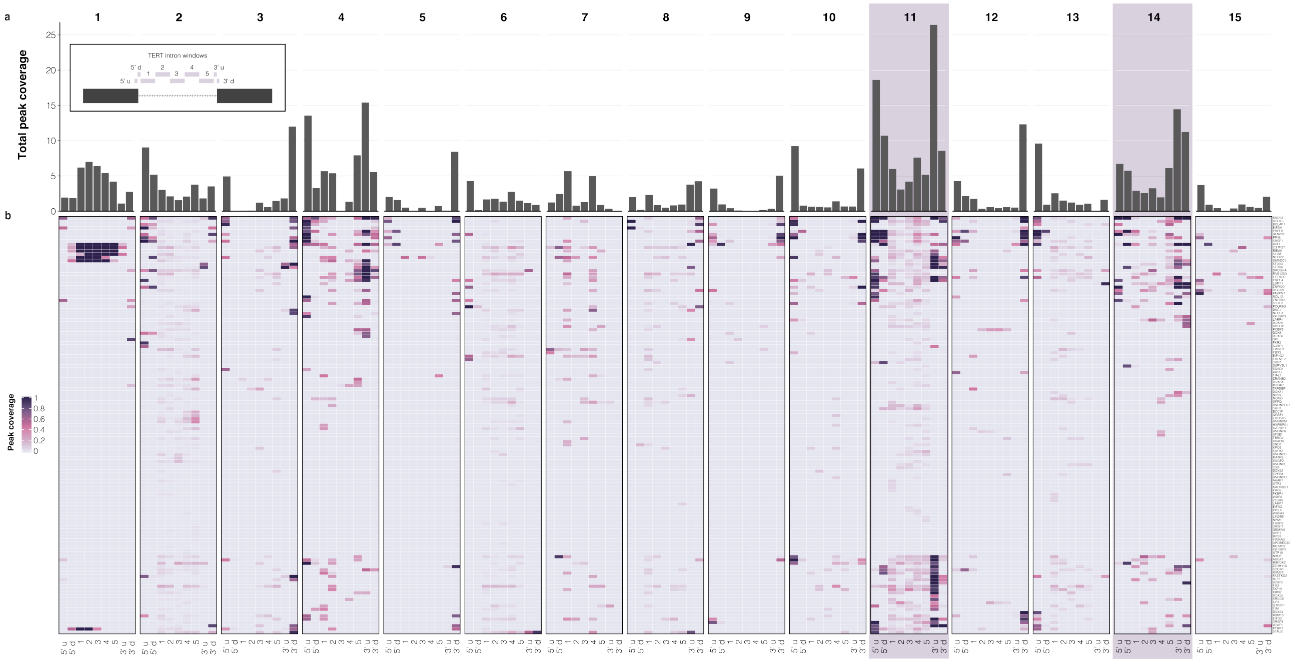

Supplementary figure 6: RNA binding protein (RBP) occupancy is increased over retained TERT introns. a, Total fraction of each intron window covered by eCLIP peaks. The 15 blocks of data represent the 15 TERT introns. Each block is then divided into windows, which are 40bp upstream and downstream of the 5’ and 3’ splice sites; intron interiors are partitioned into five windows. **b**, Heatmap colored by fraction of window covered by eCLIP peaks for each of the 127 RBPs with TERT intron binding.

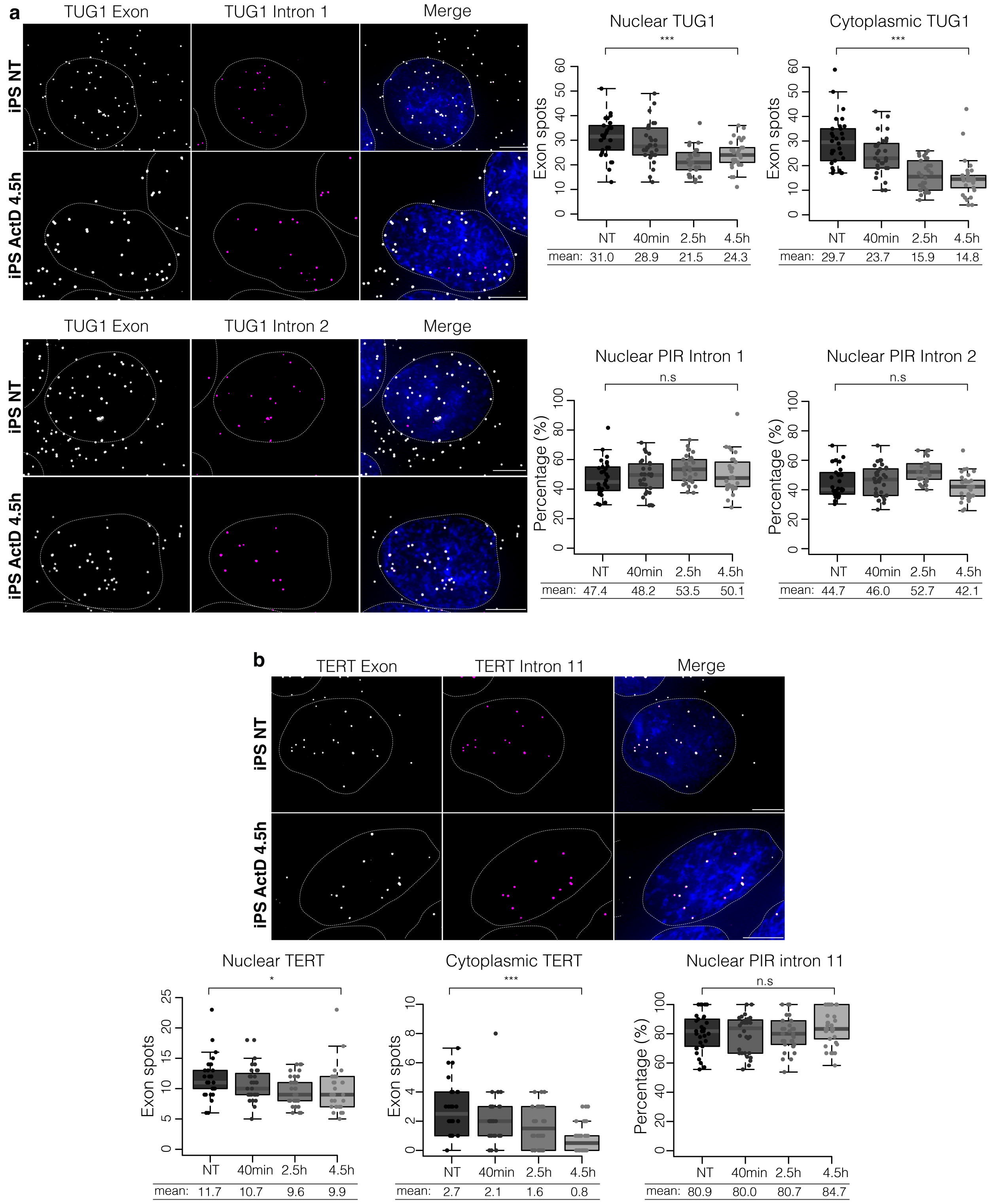

Supplementary figure 7: **a**, Maximum intensity projection of TUG1 exon and intron 1 or 2 smRNA FISH on iPS cells at time point 0 (NT) and 4.5 h after ActD treatment. On the right, quantification of spliced and unspliced TUG1 transcripts in the nucleus and cytoplasm at each time point; and percentage of nuclear intron retention (PIR) at each time point. *N* = 30 cells; for nuclear PIR intron 2 40 min, *n* = 34 cells, 2 independent measurements. **b**, Maximum intensity projection of TERT exon and intron 11 smRNA FISH on iPS cells at time point 0 (NT) and 4.5 h after ActD treatment. Below, quantification of spliced and unspliced TERT transcripts in the nucleus and cytoplasm, and percentage of nuclear intron 11 retention (PIR) at each time point. *N* (NT) = 30 cells, *n* (40 min) = 32 cells, *n* (2.5 h, 4.5 h) = 34 cells, 2 independent measurements. In **a**, **b,** midline line, median; lower and upper box limits, 25th and 75th percentiles; whiskers, 1.5 times interquartile range from 25th and 75th percentiles. *P* values were obtained by unpaired two-tailed *t*-test (equal variances), n.s. = not significant, $*P \leq 0.05$, $***P \leq 0.001$. Exon, gray; intron, magenta; DAPI, blue; scale bar, 5 µm.

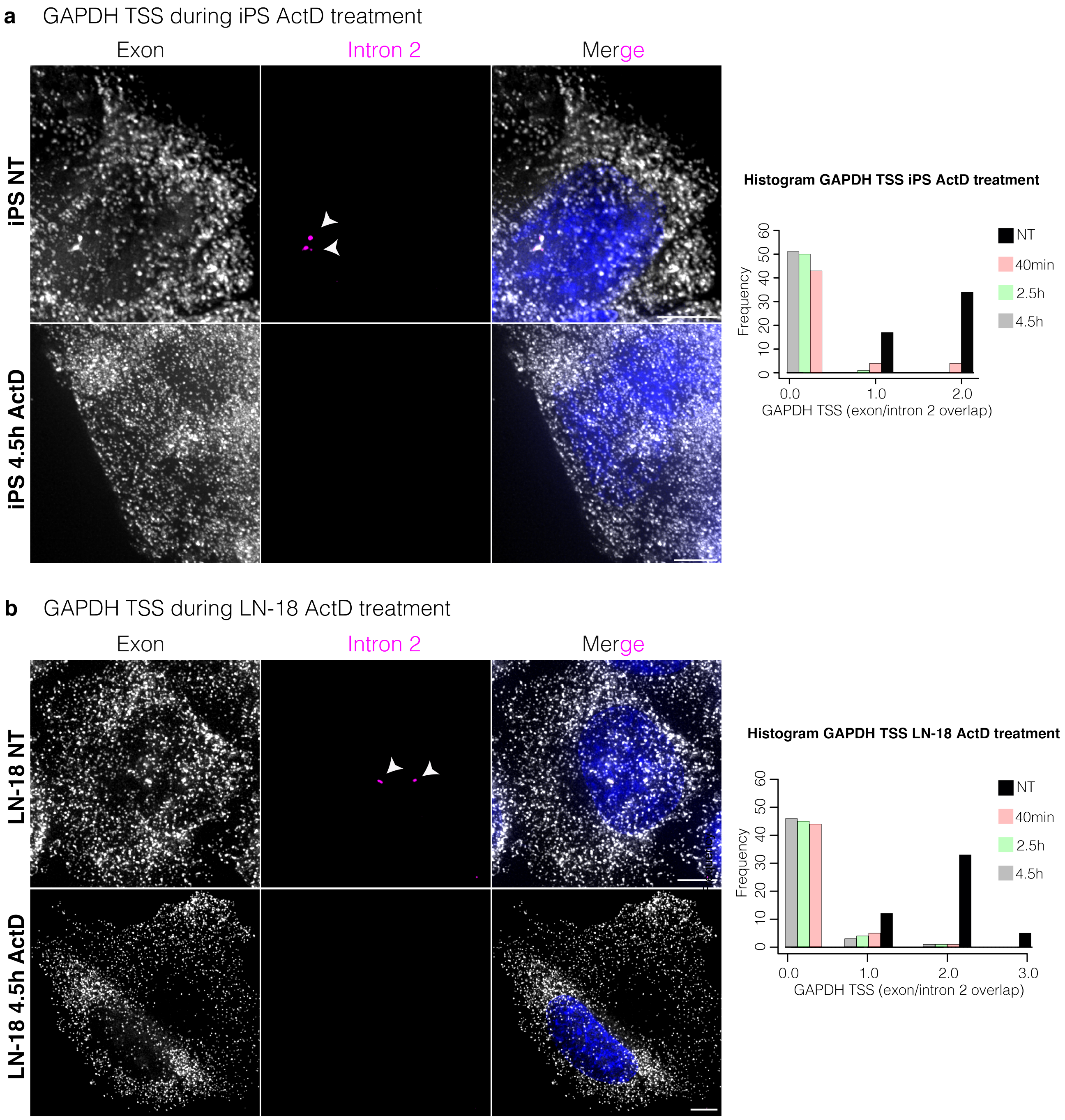

Supplementary figure 8: a, GAPDH active transcription sites (exon/intron2 overlap) during Actinomycin D (ActD) treatment of iPS cells. On the left, maximum intensity projection of GAPDH exon and intron 2 smRNA FISH on untreated cells (NT) and 4.5 h after ActD treatment. Arrowheads indicate co-localization of exon and intron signal consistent with active transcription sites. Nucleus in blue, exon in gray, intron in magenta. Scale bar, 5 µm. On the right, histogram showing the number of GAPDH transcription sites during the ActD time course. **b**, GAPDH active transcription sites (exon/intron2 overlap) during ActD treatment of LN-18 cells. On the left, maximum intensity projection of GAPDH exon and intron 2 smRNA FISH on untreated cells (NT) and after 4.5 h of ActD treatment. Arrowheads indicate co-localization of exon and intron signal consistent with active transcription sites. Nucleus in blue, exon in gray, intron in magenta. Scale bar, 5 µm. On the right, histogram showing the number of GAPDH transcription sites during the ActD time course. N = 50 cells.

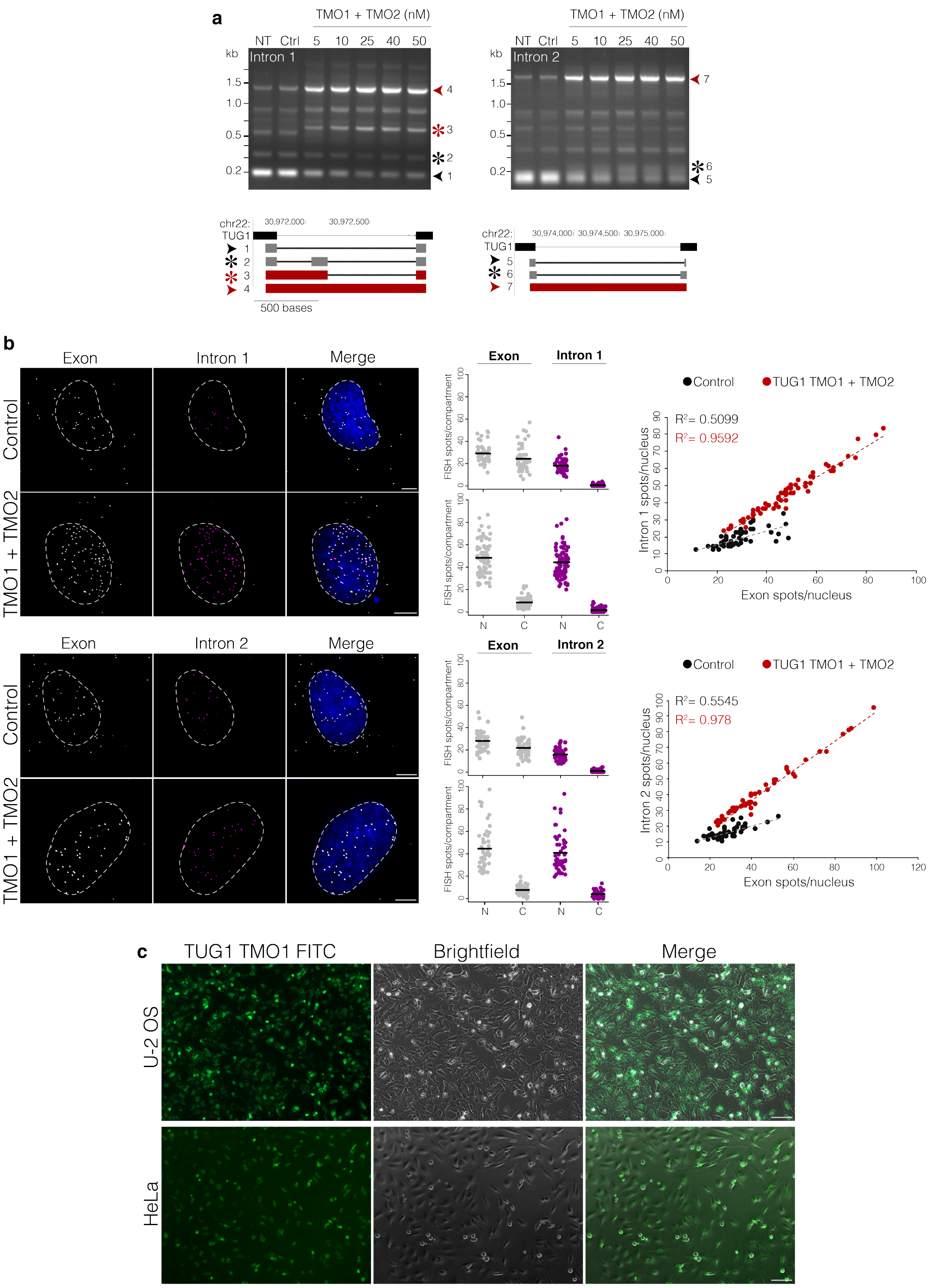

Supplementary figure 9: a, Agarose gel shown in Fig. 8e and UCSC browser displaying additional Sanger sequencing results of the intron-spanning RT PCR. Arrowheads: black, spliced product; red, unspliced product. Star: black, less abundant spliced isoform; red, unspliced form upon TMO treatment. Kb, kilobases. PCR products after transfecting TUG1 TMOs were examined on agarose gel at least three independent times. **b**, Maximum intensity projections of TUG1 exon and intron 1 or intron 2 smRNA FISH on HeLa cells transfected with control TMO and with TUG1 TMO1 and TMO2. Nucleus in blue, outlined with a dashed circle; exon, gray; intron, magenta. Scale bar, 5 µm. Middle, quantification of spliced and intron-retained TUG1 for each intron in the nucleus (N) and cytoplasm (C), solid line represents the mean. Right, correlation between nuclear exon and intron 1 and 2 in cells treated with TUG1 TMO1 and TMO2 (red) or control TMO (black). Intron 1, *n* (control) = 49 cells, *n* (TUG1 TMOs) = 81 cells; intron 2, *n* (control) = 45 cells, *n* (TUG1 TMOs) = 46 cells from two independent measurements. **c**, U-2 OS and HeLa transfection efficiency assessed by TUG1 TMO1 labeled with FITC intake (green). Scale bar, 100 µm. Monitoring of transfection efficiency with TMO FITC was performed for each transfection experiment ($n > 10$).

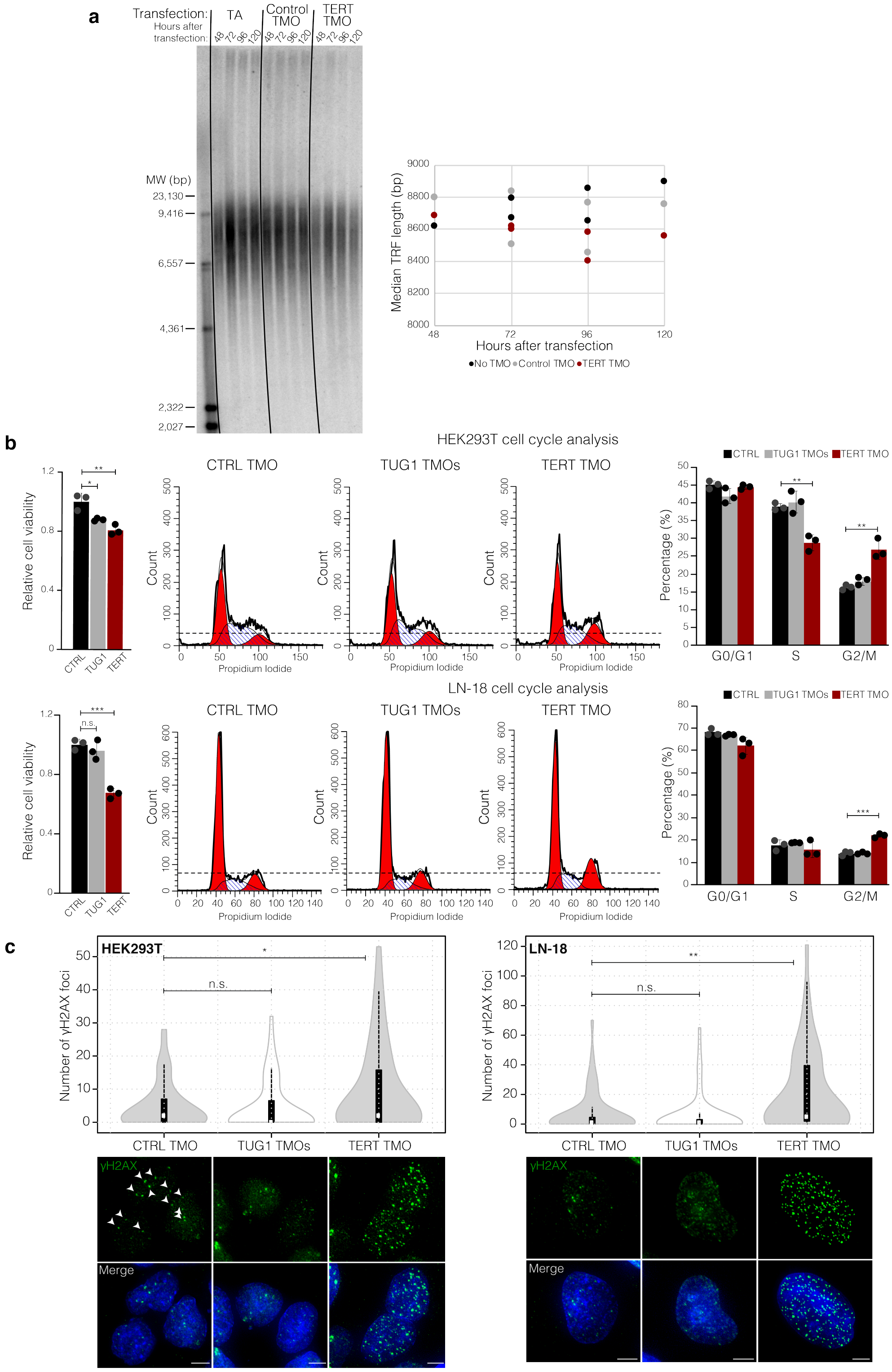

Supplementary figure 10: **a**, Telomere restriction fragment (TRF) assay of LN-18 cells treated with transfection agent only, control TMO or TERT TMO up to 120 h. Sizes are shown in base pairs, dots indicate the mean telomere size for each lane. **b**, Cell viability and cell cycle analysis of HEK293T and LN-18 cells 72 h after transfection with TUG1, TERT or control TMO relative to control TMO. Bars, means across replicates; dots, individual replicates, error bars, standard deviation of the mean of three independent measurements. **c**, Quantification of γH2A.X foci of experimental conditions shown in b. Representative images of γH2A.X immunofluorescence are shown, DAPI, blue; γH2A.X, green; scale bar 5 µm. HEK293T, *n* (control) = 52 cells, *n* (TUG1 and TERT TMOs) = 50 cells; LN-18, *n* (control) 51 cells, *n* (TUG1 and TERT TMOs) = 50 cells. White circles, median; box limits indicate the 25th and 75th percentiles; whiskers, 1.5 times the interquartile range from the 25th and 75th percentiles; polygons represent density estimates of data and extend to extreme values. For **b** and **c** *P* values were obtained by unpaired two-tailed *t*-test (equal variances), n.s. = not significant, $^{*}P \leq 0.05$, $^{**}P \leq 0.01$, $^{***}P \leq 0.001$. In **c**, adjustments for multiple comparisons were not made.

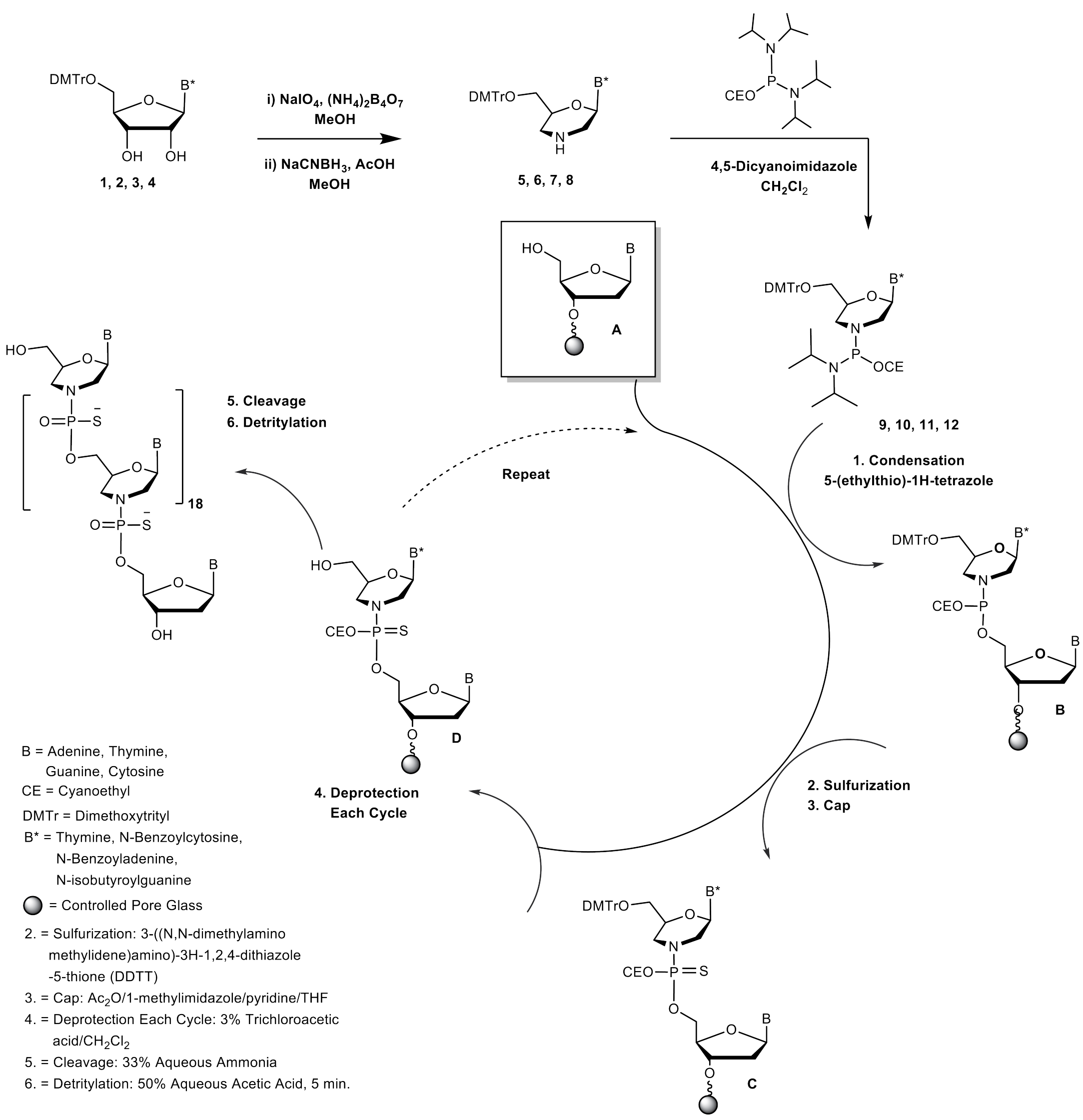

Supplementary figure 11: Synthesis of thiomorpholino oligonucleotides. Synthesis of thiomorpholino oligonucleotides. Morpholino nucleosides 5-8 and morpholino phosphorodiamidites 9-12 were synthesized starting from appropriately protected ribonucleosides 1-4 as shown in the top part of the figure. The synthesis cycle begins with detritylation of succinyl CPG 500 supported nucleoside (A). Condensation with phosphorodiamidites 9-12 generates B, which is sulfurized to produce the thiophosphoramidate morpholino triester (C). After capping the failures, detritylation is carried out to produce (D), which is then ready for the next synthesis cycle. Cleavage of final TMO oligonucleotide was carried out using 33% aqueous ammonia at 55°C for 16 h.

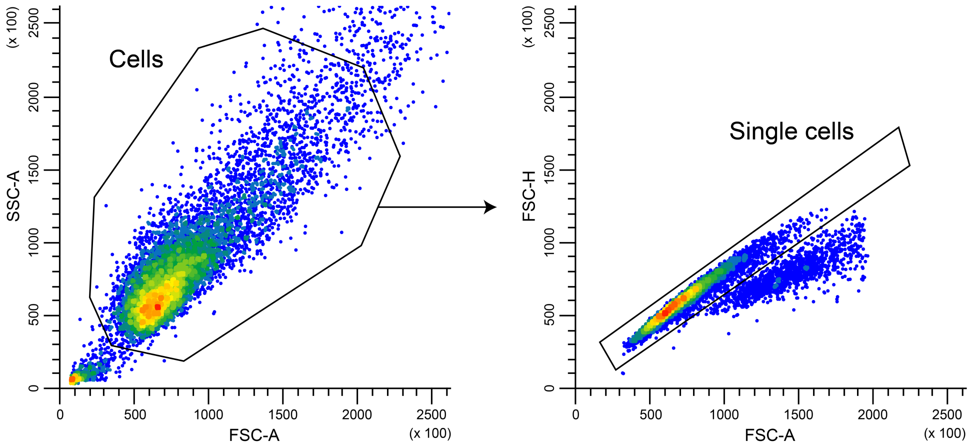

Supplementary figure 12: Gating strategy for cell cycle analysis. Cell populations were identified with SSC-A / FSC-A gate. Singlets were identified with FSC-A / FSC-H gate.
