# Supplementary material for: Nuclear compartmentalization of TERT mRNA and TUG1 lncRNA is driven by intron retention
Source: Nat Commun. 2021 Jun 3;12:3308. doi: 10.1038/s41467-021-23221-w (PMC8175569; doi:10.1038/s41467-021-23221-w)
Supplement: Supplementary file 9 — Description of additional supplementary files [file 41467_2021_23221_MOESM9_ESM.docx]

Description of additional supplementary information

Title: Supplementary data 1

Description: TERT and TUG1 PIR of each intron in hiPS, mES and miPS cells calculated with Vast-tools on RNA-Seq.

Title: Supplementary data 2

Description: TERT PIR of each intron across multiple cell and tissue types in 7 mammals.

Title: Supplementary data 3

Global PIR analysis for coding and lncRNA genes in mES and miPS cells calculated with Vast-tools on RNA-Seq.

Title: Supplementary data 4

​Global PIR analysis for coding and lncRNA genes in hiPS cells calculated with Vast-tools on RNA-Seq..

Title: Supplementary data 5

Description: Length and GC content of human and mouse retained TERT and TUG1 introns.
