# Supplementary material for: Nuclear compartmentalization of TERT mRNA and TUG1 lncRNA is driven by intron retention
Source: Nat Commun. 2021 Jun 3;12:3308. doi: 10.1038/s41467-021-23221-w (PMC8175569; doi:10.1038/s41467-021-23221-w)
Supplement: Supplementary file 10 — Reporting Summary [file 41467_2021_23221_MOESM10_ESM.pdf]

nature research

Corresponding author(s): Gabrijela, Dumbovic
John Rinn

Last updated by author(s): Mar 24, 2021

Reporting Summary

Nature Research wishes to improve the reproducibility of the work that we publish. This form provides structure for consistency and transparency in reporting. For further information on Nature Research policies, see our Editorial Policies and the Editorial Policy Checklist.

Statistics

For all statistical analyses, confirm that the following items are present in the figure legend, table legend, main text, or Methods section.

n/a	Confirmed	
☐	☒	The exact sample size (*n*) for each experimental group/condition, given as a discrete number and unit of measurement
☐	☒	A statement on whether measurements were taken from distinct samples or whether the same sample was measured repeatedly
☐	☒	The statistical test(s) used AND whether they are one- or two-sided<br>*Only common tests should be described solely by name; describe more complex techniques in the Methods section.*
☒	☐	A description of all covariates tested
☒	☐	A description of any assumptions or corrections, such as tests of normality and adjustment for multiple comparisons
☐	☒	A full description of the statistical parameters including central tendency (e.g. means) or other basic estimates (e.g. regression coefficient) AND variation (e.g. standard deviation) or associated estimates of uncertainty (e.g. confidence intervals)
☐	☒	For null hypothesis testing, the test statistic (e.g. *F*, *t*, *r*) with confidence intervals, effect sizes, degrees of freedom and *P* value noted<br>*Give P values as exact values whenever suitable.*
☒	☐	For Bayesian analysis, information on the choice of priors and Markov chain Monte Carlo settings
☒	☐	For hierarchical and complex designs, identification of the appropriate level for tests and full reporting of outcomes
☐	☒	Estimates of effect sizes (e.g. Cohen's *d*, Pearson's *r*), indicating how they were calculated

Our web collection on statistics for biologists contains articles on many of the points above.

Software and code

Policy information about availability of computer code

Data collection	Imaging: GE wide-field DeltaVision Elite microscope with an Olympus UPlanSApo 100×/1.40-numerical aperture oil objective lens and a PCO Edge sCMOS camera using appropriate filters. The built-in DeltaVision SoftWoRx Imaging software was used to deconvolve the three-dimensional stacks. Maximum intensity projections were generated with ImageJ/Fiji version 2.0.0.-rc-69/1.52p.<br>Cell viability: fluorescent data was collected using the CLARIOstar microplate reader from BMG Labtech fluorescence plate reader following the manufacturer's recommendations.<br>Southern blot: Typhoon FLA 9500 Variable Mode Imager (GE).<br>RNAseq: HiSeq2500 or HiSeq4000.<br>Public RNAseq data was downloaded from GEO.<br>Human and mouse TUG1 and TERT genomic sequences for pairwise comparison were downloaded from hg38 and mm10, respectively.
Data analysis	For the RBP analysis, motifs were retrieved from the ATtRACT database Version 0.99β. Sequence for TUG1 and TERT was retrieved using bedtools (version 2.28.0) getfasta and annotations were from Gencode v29. Motfs instance matches were calculated using FIMO from MEME version 5.1.1. eCLIP peaks were retrieved from the ENCODE project.<br>Heatmaps were generated in R version 3.6.0 using GenomicRanges (Bioconductor v. 3.10) and ComplexHeatmap version 2.2.0.<br>For intron retention analysis, raw RNA-seq reads were processed for alternative splicing and expression analysis using vast-tools, version 2.2.2 (Tapial et al., 2017), which is available on github (https://github.com/vastgroup/vast-tools), in combination with the Mm2 and Hs2 VastDB versions released on Dec. 20, 2019.<br>For generation and visualization of bam files, raw reads were mapped to GRCm38 using the nf-core RNA-seq pipeline (v1.4.2). For data analysis and visualization, R/3.4.3 or Excel/16.16.24.<br>Cell cycle analysis: ModFit LT 5.0.<br>Image analysis: ImageJ/Fiji version 2.0.0.-rc-69/1.52p.<br>Maximum entropy scores of donor and acceptor splice sites: MaxEntScan (Yeo et al., 2004).<br>TUG1 and TERT human and mouse genomic sequence alignments were prepared in Geneious using MAFFT v7.388. Pairwise sequence

comparison and visualization: CLC main workbench (Qiagen).
ChemDraw version 16.0.1.4(77) was used to make graphical illustration in Supplementary figure 11.

For manuscripts utilizing custom algorithms or software that are central to the research but not yet described in published literature, software must be made available to editors and reviewers. We strongly encourage code deposition in a community repository (e.g. GitHub). See the Nature Research guidelines for submitting code & software for further information.

Data

Policy information about availability of data

All manuscripts must include a data availability statement. This statement should provide the following information, where applicable:

- Accession codes, unique identifiers, or web links for publicly available datasets
- A list of figures that have associated raw data
- A description of any restrictions on data availability

For generating bam files, we retrieved RNA-seq data from:

HEK293: SRR3997506 [https://www.ncbi.nlm.nih.gov/sra/SRR3997506]
LN-18: SRR8769945 [https://www.ncbi.nlm.nih.gov/sra/SRR8769945]
HCT116: SRR8615282 [https://www.ncbi.nlm.nih.gov/sra/SRR8615282]
fibroblasts: SRR5420980 [https://www.ncbi.nlm.nih.gov/sra/SRR5420980]

For intron retention analysis, we used published data from human iPS cells:

GSE41716:
GSM1023087 [https://www.ncbi.nlm.nih.gov/geo/query/acc.cgi?acc=GSM1023087]
GSM1023070 [https://www.ncbi.nlm.nih.gov/geo/query/acc.cgi?acc=GSM1023070]

GSE32625:
GSM808734 [https://www.ncbi.nlm.nih.gov/geo/query/acc.cgi?acc=GSM808734]

and mouse iPS cells (GSE42100):
GSM1032506 [https://www.ncbi.nlm.nih.gov/geo/query/acc.cgi?acc=GSM1032506]
GSM1032518 [https://www.ncbi.nlm.nih.gov/geo/query/acc.cgi?acc=GSM1032518]

RNA-seq of subcellular fractions of mouse embryonic stem cells:
GSE80262 [https://www.ncbi.nlm.nih.gov/geo/query/acc.cgi?acc=GSE80262]

The data generated in this manuscript is accessible through GEO Series accession number GSE169743 [https://www.ncbi.nlm.nih.gov/geo/query/acc.cgi?acc=GSE169743], associated to figures 2, 3 and 4.

Field-specific reporting

Please select the one below that is the best fit for your research. If you are not sure, read the appropriate sections before making your selection.

☒ Life sciences ☐ Behavioural & social sciences ☐ Ecological, evolutionary & environmental sciences

For a reference copy of the document with all sections, see nature.com/documents/nr-reporting-summary-flat.pdf

Life sciences study design

All studies must disclose on these points even when the disclosure is negative.

Sample size	For RNA imaging and immunofluorescence, independent field of views were acquired (100x magnification) from at least 2 independent experiments. Approximately 50 cells were quantified for all experimental conditions while 21 to 30 cells in mitosis. This number is sufficient when the same trend is observed between cells, between independent conditions and between different cell lines.<br>Cell viability assays and cell cycle analysis were performed 3 independent times under the same conditions.<br>This is in considered sufficient to determine the reproducibility of biological effects when the same trend is observed between measurements.<br>No statistical methods were used to predetermine sample size.
Data exclusions	No data was excluded.
Replication	Reproducibility of the data was verified by performing independent biological and technical measurements.
Randomization	Imaging was performed on randomly chosen cells seeded on coverslips and random fields of view. Wells with seeded cells were randomly chosen for treatment with TMOs (or TA only).
Blinding	All experiments in any comparisons used cells grown in same conditions. The investigators were not blinded because they were designed and performed by the same person.

Reporting for specific materials, systems and methods

We require information from authors about some types of materials, experimental systems and methods used in many studies. Here, indicate whether each material, system or method listed is relevant to your study. If you are not sure if a list item applies to your research, read the appropriate section before selecting a response.

Materials & experimental systems

n/a	Involved in the study
☐	☒ Antibodies
☐	☒ Eukaryotic cell lines
☒	☐ Palaeontology and archaeology
☒	☐ Animals and other organisms
☒	☐ Human research participants
☒	☐ Clinical data
☒	☐ Dual use research of concern

Methods

n/a	Involved in the study
☒	☐ ChIP-seq
☐	☒ Flow cytometry
☒	☐ MRI-based neuroimaging

Antibodies

Antibodies used	Phospho-Histone H2A.X, Millipore Sigma 05-636, clone JBW301; Goat polyclonal Secondary Antibody to Mouse IgG - H&L (Alexa Fluor® 488), ab150113, Abcam.
Validation	Phospho-Histone H2A.X, Millipore Sigma 05-636, clone JBW301: The manufacturer validated the antibody in ChIP, ICC, IF, WB (https://www.merckmillipore.com/DE/de/product/Anti-phospho-Histone-H2A.X-Ser139-Antibody-clone-JBW301,MM_NF-05-636?ReferrerURL=https%3A%2F%2Fwww.google.com%2F).<br><br>The antibody was purchased new and used per manufacturer's instructions. No further validation was performed.<br><br>Goat polyclonal Secondary Antibody to Mouse IgG - H&L (Alexa Fluor® 488), ab150113, Abcam: The manufacturer validated the antibody in ICC/IF, Flow cytometry, IHC-Fr (https://www.abcam.com/goat-mouse-igg-hl-alexa-fluor-488-ab150113.html).<br><br>The antibody was purchased new and used per manufacturer's instructions.

Eukaryotic cell lines

Policy information about cell lines

Cell line source(s)	HCT116 (CCL-247), HeLa (CCL-2), HEK293T (CRL-3216), LN-18 (CRL-2610), U-2 OS (HTB-96) - American Type Culture Collection [ATCC]; iPSC line WTC-11 (Coriell Institute); mES (Harvard Stem Cell institute).
Authentication	The cell lines were obtained from repositories with certificate of analysis and used without further authenticated.
Mycoplasma contamination	All cell lines were tested negative for mycoplasma.
Commonly misidentified lines (See ICLAC register)	No commonly misidentified cell lines were used.

Flow Cytometry

Plots

Confirm that:

☒ The axis labels state the marker and fluorochrome used (e.g. CD4-FITC).

☒ The axis scales are clearly visible. Include numbers along axes only for bottom left plot of group (a 'group' is an analysis of identical markers).

☒ All plots are contour plots with outliers or pseudocolor plots.

☒ A numerical value for number of cells or percentage (with statistics) is provided.

Methodology

Sample preparation	Cells were washed with PBS, collected with trypsinization and spun down. Cells were fixed in 70% ethanol for at least 2 h at -20ºC. Cells were spun down; supernatant was removed and cells were resuspended in a PBS containing 35 µg/mL propidium iodide (Sigma) and 100 µg/mL RNAse A (Roche).
Instrument	Data was acquired on a BD FACSCelesta Flow Cytometer.

Software	ModFit LT 5.0 software.
Cell population abundance	10 000.
Gating strategy	Cell populations were identified with SSC-A / FSC-A gate. Singlets were identified with FSC-A / FSC- H gate.

☒ Tick this box to confirm that a figure exemplifying the gating strategy is provided in the Supplementary Information.
